# Supplementary material for: Implementation of a disability management policy in a large healthcare employer: a quasi-experimental, mixed-methods evaluation
Source: BMJ Open. 2017 Jun 17;7(6):e014734. doi: 10.1136/bmjopen-2016-014734 (PMC5541574; doi:10.1136/bmjopen-2016-014734)
Supplement: Author's manuscript [file bmjopen-2016-014734.draft_revisions.pdf]

BMJ Open

Implementation of a disability management policy in a large healthcare employer: a quasi-experimental evaluation

Journal:	*BMJ Open*
Manuscript ID	bmjopen-2016-014734
Article Type:	Research
Date Submitted by the Author:	14-Oct-2016
Complete List of Authors:	Mustard, Cameron; Institute for Work and Health,<br>Skivington, Kathryn; Glasgow University<br>Lay, Morgan; Institute for Work and Health<br>Lifshen, Marni; Institute for Work and Health<br>Etches, Jacob; Institute for Clinical Evaluative Sciences<br>Chambers, Andrea; Public Health Ontario
**Primary Subject Heading**:	Occupational and environmental medicine
Secondary Subject Heading:	Epidemiology
Keywords:	Human resource management < HEALTH SERVICES ADMINISTRATION & MANAGEMENT, Health & safety < HEALTH SERVICES ADMINISTRATION & MANAGEMENT, disability management, Canada

SCHOLARONE™
Manuscripts

For peer review only

Implementation of a disability management policy in a large healthcare employer: a quasi-experimental evaluation

Cameron A. Mustard, ScD (1,2), Kathryn Skivington, PhD (4), Morgan Lay, MPH (1), Marni Lifshen, MA (1), Jacob Etches, PhD (5), Andrea Chambers, PhD (3)

Author affiliations:
1 Institute for Work & Health, Toronto, Canada
2 Dalla Lana School of Public Health, University of Toronto, Toronto, Canada
3. Public Health Ontario, Toronto, Canada
4. University of Glasgow, Glasgow, United Kingdom
5. Institute for Clinical Evaluative Sciences, Toronto, Canada

Corresponding author:
Cameron Mustard
Institute for Work & Health
481 University Ave, Suite 800, Toronto, Ontario, Canada
T:416 927 2027, ext 2143
F: 416 927 4167
cmustard@iwh.on.ca

Key terms: human resource management, health & safety, disability management, Canada

Word count: 3,371 words

For peer review only

Abstract

Objective: This study describes the process and outcomes of the implementation of a strengthened disability management policy in a large Canadian health care employer.

Design: The study applied mixed methods, combining a process evaluation within the employer and a quasi-experimental outcome evaluation between employers for a three year period prior to and following policy implementation in January 2012.

Participants: Staff in the implementation organization (N=4,000) and staff in a peer group of 29 large hospitals (N=119,000).

Outcomes: Work disability episode incidence and duration.

Results: Both qualitative and quantitative measures of the implementation process were predominantly positive. Over the six year observation period there were 624 work disability episodes in the organization and 8,604 in the comparison group of 29 large hospitals. The annual percent change in episode incidence in the organization was -5.6 (95% CI: -9.9, -1.1) comparable to the annual percent change in the comparison group: -6.2 (-7.2, -5.3). Disability episode durations also declined in the organization, from a mean of 19.4 days (16.5, 22.3) in the pre-intervention period to 10.9 days (8.7, 13.2) in the post-intervention period. Reductions in disability durations were also observed in the comparison group: from a mean of 13.5 days (12.9, 14.1) in the 2009-2011 period to 10.5 days (9.9, 11.1) in the 2012-2014 period.

Conclusion: The incidence of work disability episodes and the durations of work disability declined strongly in this hospital sector over the six year observation period. The implementation of the organization's return-to-work policy was associated with larger reductions in disability durations than observed in the comparison group.

For peer review only - http://bmjopen.bmj.com/site/about/guidelines.xhtml

Article Summary

Strengths and limitations of this study

- There are a relatively limited number of studies evaluating the quality and effectiveness of workplace disability management programs.
- Using mixed research methods, this study reports results arising from the implementation of a strengthen disability management policy in a large health care employer in Canada.
- The evaluation design includes a comparison group of peer hospitals operating in the same jurisdiction.
- Although the evaluation design includes a quasi-experimental component, the study reports the outcomes of change in a single organization.

For peer review only

For peer review only - http://bmjopen.bmj.com/site/about/guidelines.xhtml

Introduction

This study reports on the process and outcomes of the implementation of a strengthened disability management policy in a large Canadian acute care community hospital system employing more than 4,300 staff. In collaboration with union representatives, the organization designed and implemented a return-to-work (RTW) / accommodation policy in 2012. The objective of the strengthened disability management policy was to reduce the incidence of work-related musculoskeletal disorders and improve organizational practices in the area of return-to-work and disability prevention, based on the principle that optimal workplace practices in the area of worker health will integrate efforts in the area of primary and secondary prevention (1). The outcome objectives of the strengthened disability management policy were to reduce the incidence of work disability claims registered with the provincial workers' compensation authority by 25% and to reduce the total days of disability provided wage replacement benefits by 25% over the three year period 2012-2014. The evaluation described in this study had two process evaluation elements and one outcome evaluation component: 1) a qualitative case study examining the perspectives of supervisors, managers and return-to-work coordinators regarding the fidelity of the implementation of the return-to-work / accommodation policy, 2) repeated surveys of employees who had returned to work following a disability episode and 3) a quasi-experimental design, comparing workers' compensation claim incidence and duration in the organization to a comparison group of the 29 largest acute care hospitals in the province of Ontario.

In this section, we summarize the current state of evidence for the effectiveness of workplace practices to reduce the burden of disability arising from these disorders. Three recent

For peer review only - http://bmjopen.bmj.com/site/about/guidelines.xhtml

publications (an editorial, a consensus statement and a literature review) have noted a gap in the research literature of studies evaluating the feasibility, effectiveness, costs and benefits of workplace return-to-work programs (2-4).

An early systematic review of research evidence for the effectiveness of workplace-based return-to-work (RTW) interventions found strong evidence that work disability duration is significantly reduced by work accommodation offers and contact between workplaces and workers' healthcare providers and moderate evidence that work disability duration is reduced by interventions which include early contact with worker by workplace, ergonomic work site visits, and presence of a RTW coordinator (5). For these five intervention components, there was moderate evidence that they reduce costs associated with work disability duration.

The findings of this systematic review provided specific support for core program features of workplace-based RTW programs. These core program features have been distilled to seven principles of return work (6): 1) the workplace has a strong commitment to health and safety which is demonstrated by the behaviours of the workplace parties, 2) the employer makes an offer of modified work (also known as work accommodation) to injured/ill workers so they can return early and safely to work activities suitable to their abilities, 3) RTW planners ensure that the plan supports the returning worker without disadvantaging co-workers and supervisors, 4) supervisors are trained in work disability prevention and included in RTW planning, 5) the employer makes an early and considerate contact with injured/ill workers, 6) someone has the responsibility to coordinate RTW, and 7) employers and health care providers communicate with each other about the workplace demands as needed, and with the worker's consent.

For peer review only - http://bmjopen.bmj.com/site/about/guidelines.xhtml

Franche recently reported on the factors associated with an offer of work accommodation in a cohort of more than 500 Ontario workers disabled by a musculoskeletal injury (7). Approximately 60% of workers received an offer of work accommodation in this cohort and workers were more likely to receive a work accommodation offer from their employer if the workplace was unionized or if the worker perceived strong disability prevention policies and practices in their workplace. In this large Ontario cohort, workplace factors were an important determinant of an offer of work accommodation. Workers who reported their workplace to be in the top third of a standardized measure of organizational policies and practices related to return-to-work (8-9) were three times more likely to receive an offer of work accommodation than workers who reported their workplace to be in the bottom third. Further work on this cohort has confirmed that organizational policies and practices in return-to-work are central factors in workplace disability prevention performance (10). Workers disabled by a musculoskeletal disorder who reported strong workplace organizational policies and practices in return-to-work were more likely to have returned to work at 6 months (OR=1.85; 95% CI 1.12-3.07) and at 12 months (OR=2.30; CI 1.31-4.04).

There are challenges in consistently administering a workplace disability management program (11-13). In many workplaces, an important proportion of disabled employees experience a variable and often undesirable return-to-work course including extended or intermittent work disability that results in significant individual, employer, and societal costs (13-15). Some of this inconsistency can be attributed to the sometimes competing goals of employers (e.g., minimize costs) and workers (e.g., recovery from illness), a lack of coordination and communication among work disability stakeholders (e.g., workers, health care providers, employers, and workers

For peer review only - http://bmjopen.bmj.com/site/about/guidelines.xhtml

compensation insurers), and gaps in the provision of adequate accommodations (14). In addition, supervisors and co-workers often have important responsibilities for managing different phases of the return-to-work process and their interaction with injured workers played an important role in determining work disability outcomes (16-18).

Of relevance to the objectives of this study, there is an important research literature on the factors that support successful implementation of disability management practices across organizations. An initiative in Denmark to systematically implement a national return-to-work program standard in 21 Danish municipalities identified substantial variation in disability outcomes following implementation (19-20). Evidence documented in a comprehensive process evaluation identified corresponding variation in the fidelity with which individual municipalities implemented five key dimensions of the return-to-work program and that weak implementation was associated with poor disability management outcomes (21). The current state of the research evidence concerning the effectiveness of organizational policies and practices to protect worker health points to the need for high-quality studies of the effectiveness of multi-component disability prevention programs.

The setting

The employer is one of Ontario's largest multi-site acute care community hospital systems with a $390 million annual operating budget and a staff complement of approximately 4300. Employees are represented by three unions.

For peer review only - http://bmjopen.bmj.com/site/about/guidelines.xhtml

The organization recognized limitations in the integrity of OHS policies and practices across the care sites in the system, including an under-developed disability management policy. There had been conflicts between management and representatives of workers over return-to-work practices, and the labour relations environment was frictional.

The Return-to-Work / Accommodation Policy

Over a 12 month period, the return-to-work / accommodation policy was developed jointly by the organization's management and union representatives, with contributions from not-for-profit external advisors. The policy was closely anchored to guidance provided by research evidence (5, 22-23), including an emphasis on early contact, the designation of disability case managers, the integration of supervisors in the development of return-to-work plans and the provision of education and training to managers and supervisors. A distinctive component of the policy was a specific role defined for union representatives as 'Return-to-Work Coordinators' (RTWC)(24). RTWCs participated in return-to-work planning meetings, along with the employee, the organization's disability case managers and the employee's supervisor. Implementation of the renewed return-to-work / accommodation policy commenced in January 2012.

The outcome objectives of the strengthened disability management policy were to reduce the incidence of work disability claims registered with the provincial workers' compensation authority by 25% and to reduce the total days of disability provided wage replacement benefits by 25% over the three year period 2012-2014.

For peer review only - http://bmjopen.bmj.com/site/about/guidelines.xhtml

Methods

Study Design

This study applied a mixed methods protocol consisting of two components. The process evaluation component had two elements: 1) a qualitative study of the perspectives of managers and supervisors concerning the fidelity of the implementation of the return to work / accommodation policy and, 2) the administration of two annual surveys of a sample of NHS staff who had returned to work following a disability episode. The outcome evaluation component applied a quasi-experimental design, comparing workers' compensation claim incidence and duration in the organization to a peer group of 29 large Ontario hospitals for a three year period prior to and following January 2012.

Process evaluation: supervisors and return-to-work coordinators

All managers and RTWCs who had been involved in a RTW episode in 2012 (N=104) were invited to participate in in-person semi-structured interviews. Interviews were completed with 21 managers and nine union RTWCs. Interviews followed a topic guide with general themes about the RTW policy: roles and responsibilities under the process; early contact practices; how the RTW plan was developed; how RTW is monitored; access to confidential information; and general overall assessment of the positive and negative elements of the program. A thematic analysis of interview transcripts was performed.

Process evaluation: Employees experiencing a disability episode

All employees who participated in a return-to-work plan following a disability episode in 2013 and 2014 were invited to complete an internet-administered 14 item questionnaire. In both years,

For peer review only - http://bmjopen.bmj.com/site/about/guidelines.xhtml

the response rate was approximately 30%. The 14 item questionnaire asked respondents for their perception of the accommodation process using a five-point Likert scale (strongly agree to strongly disagree). Respondents were also invited to contribute free text responses on their overall experience, initial contact with the NHS's occupational health and safety staff, the RTW transition, and issues around health information confidentiality. Chi-square tests were applied to test for differences between 2013 and 2014 in the proportion of respondents who endorsed each of the 14 questionnaire items.

Outcome evaluation:

The outcome evaluation component applied a quasi-experimental design, comparing workers' compensation claim incidence and duration in the organization to a peer group of the 29 largest Ontario hospitals over the six year period 2009-2014.

Electronic abstracts of administrative records of lost-time and no lost-time compensation claims were obtained from the Workplace Safety & Insurance Board (WSIB), the publicly-administered, single-payer work disability insurer in Ontario. The outcome evaluation component of this study is based on two measures obtained from administrative data: 1) the incidence rate of lost-time and no lost-time claims and 2) the mean duration of lost-time disability episodes arising from work-related disorders. The incidence rates for lost-time and no lost-time claims were computed on an annual basis with denominator information of person years of employment obtained from WSIB insurance records of the organization and the peer group of 29 large hospitals. The duration of disability was computed for each lost-time claim based on days of wage replacement benefits and the mean duration of disability, in days,

For peer review only - http://bmjopen.bmj.com/site/about/guidelines.xhtml

computed for all claims on an annual basis. Confidence intervals (95%) for incidence rates and disability day rates were estimated under an assumption of a Poisson distribution. We estimated average annual percent change in incidence rates using negative binomial regression with the log of person-years of employment as an offset. We estimated annual rates of return-to-work at intervals of seven, fourteen, thirty and sixty days using piecewise exponential survival models in SAS PROC GENMOD and tested for differences in trends of return-to-work durations between the organization and the peer group (25). The outcome evaluation component of this study was designed to have sufficient power to detect a reduction in the incidence of workers' compensation claims from 75 per 1000 full-time equivalents (2009-2011) to 65 per 1000 full-time equivalents over the period 2012-2014.

Ethics

Ethical approval was obtained from the Health Sciences Research Ethics Board, University of Toronto (Protocol 28503).

For peer review only - http://bmjopen.bmj.com/site/about/guidelines.xhtml

Results

The implementation of the return-to-work / accommodation policy was the responsibility of the organization's occupational health and safety (OHS) department. The progress of the implementation was monitored by a labour-management steering committee, chaired by the organization's vice-president, human resources. Steering committee membership include union local leadership, OHS department staff and external members from a number of provincial labour unions. The steering committee met 4-6 times per year during the three year implementation period documented in this study. The research team was also a member of the steering committee and periodically provided reports on aspects of the process and outcomes of the policy implementation.

Process evaluation component

Both qualitative and quantitative measures of the implementation process were predominantly positive. Supervisors, managers and RTWCs who participated in semi-structured interviews in 2013 noted a number of achievements (24). Informants offered the view that occupational health and safety practice had improved since the implementation of the policy and spoke of the clear internal communication around the return-to-work / accommodation policy to all levels of staff in the organization. Informants also perceived a renewed emphasis on returning disabled workers to their pre-injury positions as quickly as possible and that the staff in the occupational health and safety department were well respected. There was recognition that the quality and consistency of disability management practices were improving, that the collaboration between the employer and the labour unions was strengthened and that the return-to-work planning

For peer review only - http://bmjopen.bmj.com/site/about/guidelines.xhtml

meeting was an important opportunity for establishing clarity for the goals of individual return-to-work plans.

Managers, supervisors and return-to-work coordinators also identified a number of opportunities for improvement. Informants encouraged a greater emphasis on timely access to information about the status of individual disability episodes, and identified a number of opportunities to improve the consistency of case management across disability episodes. Informants also described a number of areas where the roles and responsibilities return-to-work coordinators and supervisors were ambiguous and emphasized the importance of providing orientation to newly-appointed personnel. Interview participants also acknowledged that the OHS department had a high case load and constrained resources.

For peer review only

Employees experiencing a disability episode

Approximately 30% of employees who participated in a return to work plan accepted the invitation to complete an internet-administered 14 item questionnaire. The majority of respondents who returned to work in 2013 (N=78) and in 2014 (N=54) reported positive assessments of the return-to-work process (Table 1). Employees gave strong endorsement to the purpose and outcome of the return-to-work planning meeting and expressed satisfaction with the protection of personal health information. Respondents in 2014 reported stronger endorsement of a number of program dimensions compared to respondents in 2013. For example, a higher proportion of respondents in 2014 were satisfied with modified duty arrangements and a smaller proportion of respondents in 2014 reported delays in arranging the initial return-to-work planning meeting. There were no significant differences in perceptions of the return-to-work

For peer review only - http://bmjopen.bmj.com/site/about/guidelines.xhtml

process between respondents whose disability was attributed to a work-related cause, and respondents whose disability was attributed to a non-occupational cause.

Free text responses provided by survey respondents were approximately balanced between positive and negative statements. Themes that were emphasized in the free text responses included the importance of supportive, compassionate and respectful interactions with OHS staff, the importance of support from managers and supervisors and the value of efficient and timely communication with OHS staff. Respondents also emphasized the value of monitoring the progress of accommodations over the course of the disability episode and the importance of aligning modified duties to clinical recommendations.

<u>Outcome evaluation component</u>

Over the six year observation period there were 1,916 total compensation claims in the organization, and 29,974 compensation claims in the comparison group of 29 large hospitals (Table 2). The incidence rates for lost-time claims and for no lost-time claims were substantially higher in the organization relative to the comparison group. In both the organization and the comparison group, the decline in the incidence of lost-time claims between the 2009-2011 period and the 2012-2014 period was statistically significant. The annual percent change in the lost-time claim incidence rate in the organization was -5.6 (95% CI: -9.9, -1.1) comparable to the annual percent change in the comparison group: -6.2 (-7.2, -5.3).

Disability durations also declined in the organization, from a mean of 19.4 days (16.5, 22.3) in the pre-intervention period to 10.9 days (8.7, 13.2) in the post-intervention period (Table 3).

For peer review only - http://bmjopen.bmj.com/site/about/guidelines.xhtml

Reductions in disability durations were also observed in the comparison group: from a mean of 13.5 days (12.9, 14.1) in the 2009-2011 period to 10.5 days (9.9, 11.1) in the 2012-2014 period.

In the pre-intervention period, the proportion of disability episodes in the organization that had returned to work at seven days, 30 days and 60 days was substantially lower than observed outcomes in the peer group. As depicted in Figure 1, the proportion of disability episodes that had returned to work at seven days improved in both the organization and the comparison group over the six year observation period, with a stronger improvement in the organization. A test for difference in the trend of improvement between the organization and the comparison group approached statistical significance (p=.07).

For peer review only

For peer review only - http://bmjopen.bmj.com/site/about/guidelines.xhtml

Discussion

This study has described the process and outcomes of a three year effort to implement a strengthened disability management policy in a large healthcare employer. Both the process and outcome findings of this study document a successful implementation of the strengthened policy. Managers, supervisors and return-to-work coordinators broadly endorsed the strengthened policy. Employees who returned to work following a disability episode provided predominantly positive assessments across all dimensions of the disability management process. While the annual percent reduction in lost-time claims in the organization was similar to the reductions observed in a peer group of 29 hospitals over a six year period, the implementation of the organization's strengthened return-to-work policy was associated with greater improvements in disability durations than observed in the peer group.

Despite the broad consensus that workplace polices and practices are a central determinant of return-to-work outcomes, the literature on the implementation of disability management programs in individual workplaces is limited and represents a research gap (2-3). This study contributes to addressing this research gap. While the observational study was not suitable for determining which elements of the strengthened disability management policy were most responsible for the improvements in disability durations, the favourable process and outcome measures reported in this study indicate that it is feasible to successfully implement a moderately complex multi-component disability management policy. From the perspective of employees who returned to work following a disability episode, it appears that all elements of the return-to-work process were meaningful, including early contact, the process and outcome of the return-to-work planning meeting and the support of supervisors and union representatives in the transition

For peer review only - http://bmjopen.bmj.com/site/about/guidelines.xhtml

back to work. The collaborative model adopted by this organization, where representatives of a worker's union participate in return-to-work planning, appears to have functioned effectively. RTW coordinators and disability management staff developed respect for the consistency and transparency of the collaborative process. And we note there was continuity and consistency in the organizational leadership supporting this initiative.

The evaluation design combining qualitative and quantitative methods to document the process and outcome of the policy implementation was a strength of this study. The inclusion of a comparison group of peer hospitals operating in the same geographic jurisdiction provided an accurate calibration of the magnitude of change achieved by the organization.

We would briefly note the following limitations. Although the evaluation design did include a quasi-experimental component, the study has reported on the process and outcome of change in a single organization. Stronger insights into the facilitators and barriers of organizational improvement in disability management practices would be available from evaluations of implementation process and outcome across multiple workplaces, where the attributes of organizations that successfully accomplish the program objective can be compared to organizations that are less successful (21, 26-27). While this study has compared the incidence and durations of disability episodes arising from work-related conditions between the organization and a large hospital peer-group, we were unable to acquire similar administrative records of work disability incidence and duration for disability episodes not attributed to work exposures.

For peer review only

The organizational change initiative reported in this study was designed jointly by labour and management representatives within a large Ontario public sector employer, with technical assistance provided by not-for-profit external agencies with expertise in workplace quality improvement initiatives. This organization change initiative was accomplished with the technical and human resources available to the employer and did not require significant external resources. Relative to the outcome objectives established at the initiation of this quality improvement initiative, the organization accomplished a reduction of approximately 15% in the incidence of work disability claims and achieved an approximate 50% reduction in average disability episode durations over the three year period following the implementation of the strengthen disability management policy. Replication of the collaborative model described in this study may be considered in other healthcare settings in this jurisdiction.

For peer review only

Contributorship statement

C Mustard conceptualized and designed the study, interpreted the data and wrote the first draft of the paper. K Skivington, M Lifshen, A Chambers and M Lay contributed to data collection and data interpretation. J Etches contributed to the analysis of the study data and provided interpretation. All authors contributed to the review and revision of the paper.

Competing interests

The authors declare no competing interests.

Funding

Funding support for the study reported in this paper was provided by the Canadian Institute of Health Research (Grant No. 123365). The Institute for Work & Health operates with support from the Government of Ontario.

Data sharing statement

No additional data available.

For peer review only - http://bmjopen.bmj.com/site/about/guidelines.xhtml

References

1. Frank J, Cullen K. Preventing injury, illness and disability at work. Scandinavian Journal of Work, Environment and Health. 2006;32(2):160-7.

2. Viikari-Juntura E, Burdorf A. Return to work and job retention – increasingly important outcomes in occupational health research. Scand J Work Environ Health. 2011;37(2):81-84.

3. Vanderbroeck S, Verjans M, Lambreghts C, Godderis L. Research review on rehabilitation and return to work. Luxembourg: European Agency for Safety and Health at Work; 2016. doi: 978-92-9240-924-1

4. Main CJ, Shaw WS; Hopkinton Conference Working Group on Workplace Disability Prevention. Employer Policies and Practices to Manage and Prevent Disability: Conclusion to the Special Issue. J Occup Rehabil. Epub 2016 Jul 30. PubMed PMID: 27475446.

5. Franche RL, Cullen K, Clarke J, Irvin E, Sinclair S, Frank J. Workplace-based return-to-work interventions: a systematic review of the quantitative literature. Journal of Occupational Rehabilitation. 2005;15(4):607-31.

6. Institute for Work & Health. Seven 'principles' for successful return to work. Available from: http://www.iwh.on.ca/files/seven_principles_rtw_2007.pdf.

7. Franche RL, Severin C, Hogg-Johnson S, Lee H, Côté P, Krause N. Multivariate analysis of factors associated with early offer and acceptance of a work accommodation following an occupational musculoskeletal injury. Journal of Occupational and Environmental Medicine. 2009 Aug;51:969-983.

8. Williams RM, Westmorland MG, Shannon HS, Amick BC. Disability management practices in Ontario health care workplaces. J Occup Rehabil. 2007 Mar;17(1):153-65.

9. Williams RM, Westmorland MG, Shannon HS, Rasheed F, Amick BC. Disability management practices in education, hotel/motel and health care workplaces. Am J Ind Med 2005;47:217–26.

10. Amick BC, Steenstra I, Hogg-Johnson S, Katz JN, Lee H, Brouwer S, Franche RL, Bultmann U. How do organizational policies and practices affect return-to-work and work role functioning following a musculoskeletal injury. J Occup Rehabil. Forthcoming.

11. Costa-Black KM, Feuerstein M, Loisel P. Work Disability Models: Past and Present. In: Handbook of Work Disability. New York: Springer; 2013. p. 71-93.

12. Amick BC, Habeck R, Hunt A, Fossel A, Chapin A, Keller R, et al. Measuring the impact of organizational behaviors on work disability prevention and management. Journal of Occupational Rehabilitation. 2000;10(1):21-38.

13. MacEachen E, Kosny A, Ferrier S, Chambers L. The "toxic dose" of system problems: why some injured workers don't return to work as expected. Journal of Occupational Rehabilitation. 2010;20(3):349-66.

For peer review only

14. MacEachen E, Kosny A, Ferrier S. Unexpected barriers in return to work: lessons learned from injured worker peer support groups. Work: A Journal of Prevention, Assessment and Rehabilitation. 2007;29(2):155-64.

15. Jetha A, Pransky G, Fish J, Hettinger LJ. Return-to-work within a complex and dynamic organizational work disability system. J Occup Rehabil. 2016 Sep;26(3):276-85. doi: 10.1007/s10926-015-9613-2.

16. Besen E, Young AE, Shaw WS. Returning to work following low back pain: towards a model of individual psychosocial factors. Journal Occup Rehabil. 2014:1-13.

17. Tjulin Å, MacEachen E, Ekberg K. Exploring workplace actors experiences of the social organization of return-to-work. Journal Occup Rehabil. 2010;20(3):311-21.

18. Linton S, Boersma K, Traczyk M, Shaw W, Nicholas M. Early workplace communication and problem solving to prevent work disability: results of a randomized controlled trail among high-risk workers and their supervisors. J Occup Rehabil. 2016;26:150-159.

19. Aust B, Helverskov T, Nielsen MB, Bjorner JB, Rugulies R, Nielsen K, et al. The Danish national return-to-work program – aims, content, and design of the process and effect evaluation. Scand J Work Environ Health. 2012 Mar;38(2):120-33. doi: 10.5271/sjweh.3272.

20. Nielsen MB, Vinsløv Hansen J, Aust B, Tverborgvik T, Thomsen BL, et al. A multisite randomized controlled trial on time to self-support among sickness absence beneficiaries. The Danish national return-to-work programme. Eur J Public Health. 2015 Feb;25(1):96-102. doi: 10.1093/eurpub/cku016.

21. Aust B, Nielsen MB, Grundtvig G, Buchardt HL, Ferm L, Andersen I, et al. Implementation of the Danish return-to-work program: process evaluation of a trial in 21 Danish municipalities. Scand J Work Environ Health. 2015 Nov;41(6):529-41. doi:10.5271/sjweh.3528.

22. Pranksy G, Shaw W, Loisel P, Hong N.H, Désorcy B. Development and validation of competencies for Return to Work Coordinators. J Occup Rehabil. 2010;20:41-48.

23. Gensby U, Labriola M, Irvin E, Amick BC, Lund T. A classification of components of workplace disability management programs: results from a systematic review. J Occup Rehabil. 2014 Jun;24(2):220-41. doi: 10.1007/s10926-013-9437-x.

24. Skivington K, Lifshen M, Mustard CA. Implementing a collaborative return-to-work programme: lessons from a case study of a large employer in Canada. Work. Forthcoming.

25. Allison D. Survival analysis using SAS: A practical guide. 2nd ed. SAS Institute; 2010.

26. Fassier JB, Durand MJ, Caillard JF, Roquelaure Y, Loisel P. Results of a feasibility study: barriers and facilitators in implementing the Sherbrooke model in France. Scand J Work Environ Health. 2015 May 1;41(3):223-33. doi:10.5271/sjweh.3489.

27. Chambers A, Mustard CA, Holness LD, Nichol K, Breslin CF. Barriers to the adoption of safety-engineered needles following a regulatory standard: Lessons learned from three acute care hospitals. Healthcare Policy. 2015;11(1):90-101.

For peer review only

For peer review only - http://bmjopen.bmj.com/site/about/guidelines.xhtml

Table 1
Returned-to-work employees' perceptions of the disability management process
Five point likert scale (strongly disagree to strongly agree)
Highlighted items indicate significant difference (improvement) between 2013 and 2014 surveys

	Percent of employees agreeing with statement 2013 (n=78)	2014 (n=54)	Chi sq
**Supported through the process:**			
My supervisor/manager supported my transition back to work	70%	76%	0.49
My coworkers supported by transition back to work	76%	78%	0.78
My absence and my return-to-work experience was in keeping with my employer's core values of compassion, professionalism and respect	53%	67%	0.11
**Satisfaction with the process:**			
Overall, I was satisfied with my union's support in my return-to-work	72%	69%	0.69
Overall, I was satisfied with the employer's support in my return-to-work	58%	68%	0.21
I was satisfied with arrangement to modify my job duties and/or work hours during the transition back to full regular duties	60%	81%	0.01
**Confidence about the process:**			
Timeliness			
There were delays in arranging my return-to-work planning meeting	42%	28%	0.09
Documentation from my healthcare provider supported the timeliness of my return-to-work	83%	91%	0.22
I was contacted shortly after my absence began by the Occupational Health and Safety Department	76%	80%	0.60
The return-to-work meeting			
I understood the purpose of the return-to-work planning meeting	87%	87%	0.98
The return-to-work planning meeting identified options to modify or redesign my regular work and/or hours	67%	83%	0.03
I was an active participant in the return-to-work planning meeting	79%	82%	0.77
Planning my return-to-work was a team effort	64%	80%	0.05
Confidentiality			
Confidential information about my health was protected	72%	85%	0.07

For peer review only - http://bmjopen.bmj.com/site/about/guidelines.xhtml

Figure 1
Trend in disability benefit duration, seven days or less
Organization compared to 29 peer hospitals, Ontario 2009-2014
Work-related lost-time compensation claims

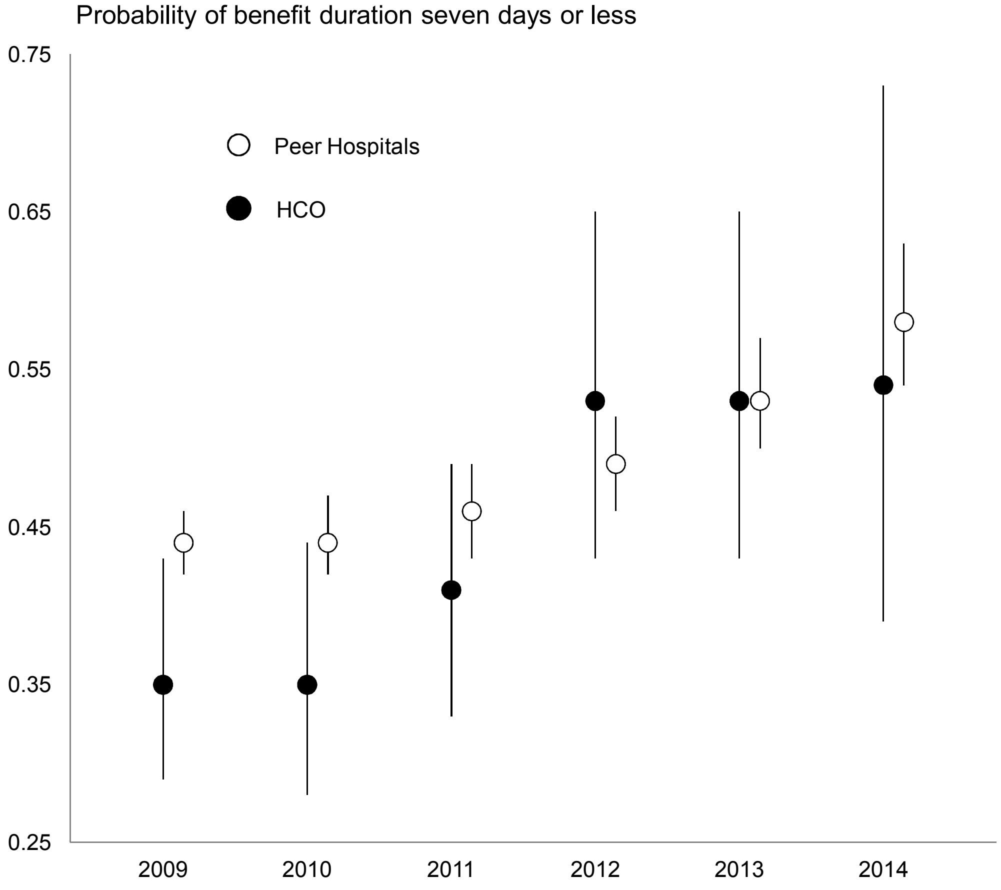

only

For peer review only - http://bmjopen.bmj.com/site/about/guidelines.xhtml

Table 2
Incidence of compensation claims, Organization compared to 29 peer hospitals, 2009-2014

	2009-2011				2012-2014			
	N	FTE	Rate/1000	95% CI (1)	N	FTE	Rate/1000	95% CI (1)
**Lost-time claims**								
Organization	362	12,062	30.0	27.1 - 33.3	287	12,849	22.3	19.9 - 25.1
Peer Hospitals	4,983	357,104	14.0	13.6 - 14.3	3,986	379,150	10.5	10.2 - 10.8
**No Lost-time claims**								
Organization	631	12,062	52.3	48.4 - 56.6	636	12,849	49.5	45.8 - 53.5
Peer Hospitals	9,835	357,104	27.5	27.0 - 28.1	11,170	379,150	29.5	28.9 - 30.0
**Total claims**								
Organization	993	12,062	82.3	77.4 - 87.6	923	12,849	71.8	67.3 - 76.6
Peer Hospitals	14,818	357,104	41.5	40.8 - 42.2	15,156	379,150	40.0	39.3 - 40.6

(1) The CI assumes a Poisson distribution in the numerator.

ew only

For peer review only - http://bmjopen.bmj.com/site/about/guidelines.xhtml

Table 3
Lost-time compensation claim benefit duration, Organization compared to 29 peer hospitals, 2009-2014
Average benefit days per claim, first 90 days post-injury

	2009-2011			2012-2014		
	Number of Claims	Average benefit days	95% CI	Number of Claims	Average benefit days	95% CI
**Lost-time claims**						
Organization	362	19.4	16.5 - 22.3	287	10.9	8.7 - 13.2
Peer Hospitals	4,983	13.5	12.9 - 14.1	3,986	10.5	9.9 - 11.1

peer review only

For peer review only - http://bmjopen.bmj.com/site/about/guidelines.xhtml

BMJ Open

Implementation of a disability management policy in a large healthcare employer: a quasi-experimental, mixed-methods evaluation

Journal:	*BMJ Open*
Manuscript ID	bmjopen-2016-014734.R1
Article Type:	Research
Date Submitted by the Author:	20-Apr-2017
Complete List of Authors:	Mustard, Cameron; Institute for Work and Health,<br>Skivington, Kathryn; Glasgow University<br>Lay, Morgan; Institute for Work and Health<br>Lifshen, Marni<br>Etches, Jacob; Institute for Clinical Evaluative Sciences<br>Chambers, Andrea; Public Health Ontario
**Primary Subject Heading**:	Occupational and environmental medicine
Secondary Subject Heading:	Epidemiology
Keywords:	Human resource management < HEALTH SERVICES ADMINISTRATION & MANAGEMENT, Health & safety < HEALTH SERVICES ADMINISTRATION & MANAGEMENT, disability management, Canada

SCHOLARONE™
Manuscripts

For peer review only

IWH Project 1250

Submitted for review, October 13, 2016: BMJ Open

Revision: April 12, 2017

Implementation of a disability management policy in a large healthcare employer: a quasi-experimental, mixed-methods evaluation

Cameron A. Mustard, ScD (1,2), Kathryn Skivington, PhD (4), Morgan Lay, MPH (1), Marni Lifshen, MA (1), Jacob Etches, PhD (5), Andrea Chambers, PhD (3)

Author affiliations:
1 Institute for Work & Health, Toronto, Ontario
2 Dalla Lana School of Public Health, University of Toronto, Toronto, Ontario
3. Public Health Ontario
4. University of Glasgow
5. Institute for Clinical Evaluative Sciences

Corresponding author:
Cameron Mustard
Institute for Work & Health
481 University Ave, Suite 800, Toronto, Ontario, Canada
416 927 2027, ext 2143
cmustard@iwh.on.ca

Running head: Evaluation of the implementation of a disability management policy

Key terms: disability management; workplace intervention; injury prevention; Canada

For peer review only

Abstract

Objective: This study describes the process and outcomes of the implementation of a strengthened disability management policy in a large Canadian health care employer. Key elements of the strengthened policy included an emphasis on early contact, the training of supervisors and the integration of union representatives in return-to-work planning.

Design: The study applied mixed methods, combining a process evaluation within the employer and a quasi-experimental outcome evaluation between employers for a three year period prior to and following policy implementation in January 2012.

Participants: Staff in the implementation organization (N=4,000) and staff in a peer group of 29 large hospitals (N=119,000).

Outcomes: Work disability episode incidence and duration.

Results: Both qualitative and quantitative measures of the implementation process were predominantly positive. Over the six year observation period there were 624 work disability episodes in the organization and 8,604 in the comparison group of 29 large hospitals. The annual percent change in episode incidence in the organization was -5.6 (95% CI: -9.9, -1.1) comparable to the annual percent change in the comparison group: -6.2 (-7.2, -5.3). Disability episode durations also declined in the organization, from a mean of 19.4 days (16.5, 22.3) in the pre-intervention period to 10.9 days (8.7, 13.2) in the post-intervention period. Reductions in disability durations were also observed in the comparison group: from a mean of 13.5 days (12.9, 14.1) in the 2009-2011 period to 10.5 days (9.9, 11.1) in the 2012-2014 period.

Conclusion: The incidence of work disability episodes and the durations of work disability declined strongly in this hospital sector over the six year observation period. The implementation of the organization's return-to-work policy was associated with larger reductions in disability durations than observed in the comparison group.

For peer review only - http://bmjopen.bmj.com/site/about/guidelines.xhtml

Strengths and limitations of this study

A mixed-methods evaluation of the process and outcomes of the implementation of a disability management policy in a large employer

The evaluation includes a quasi-experimental design, comparing temporal changes in outcomes in the intervention organization to a group of peer organizations

Stronger insights into the facilitators and barriers to organizational improvement would be available from the evaluation of implementation across multiple workplaces

For peer review only

For peer review only - http://bmjopen.bmj.com/site/about/guidelines.xhtml

Introduction

This study reports on the process and outcomes of the implementation of a strengthened disability management policy in a large Canadian acute care community hospital system employing more than 4,300 staff. In collaboration with union representatives, the organization designed and implemented a return-to-work (RTW) / accommodation policy in 2012. The objective of the strengthened disability management policy was to reduce the incidence of work-related musculoskeletal disorders and improve organizational practices in the area of return-to-work and disability prevention, based on the principle that optimal workplace practices in the area of worker health will integrate efforts in the area of primary and secondary prevention (1). The outcome objectives of the strengthened disability management policy were to reduce the incidence of work disability claims registered with the provincial workers' compensation authority by 25% and to reduce the total days of disability provided wage replacement benefits by 25% over the three year period 2012-2014. The evaluation described in this study had two process evaluation elements and one outcome evaluation component: 1) a qualitative case study examining the perspectives of supervisors, managers and return-to-work coordinators regarding the fidelity of the implementation of the return-to-work / accommodation policy, 2) repeated surveys of employees who had returned to work following a disability episode and 3) a quasi-experimental design, comparing workers' compensation claim incidence and duration in the organization to a comparison group of the 29 largest acute care hospitals in the province of Ontario.

In this section, we summarize the current state of evidence for the effectiveness of workplace practices to reduce the burden of disability arising from these disorders. Three recent

For peer review only - http://bmjopen.bmj.com/site/about/guidelines.xhtml

publications (an editorial, a consensus statement and a literature review) have noted a gap in the research literature of studies evaluating the feasibility, effectiveness, costs and benefits of workplace return-to-work programs (2-4).

An early systematic review of research evidence for the effectiveness of workplace-based return-to-work (RTW) interventions found strong evidence that work disability duration is significantly reduced by work accommodation offers and contact between workplaces and workers' healthcare providers and moderate evidence that work disability duration is reduced by interventions which include early contact with worker by workplace, ergonomic work site visits, and presence of a RTW coordinator (5). For these five intervention components, there was moderate evidence that they reduce costs associated with work disability duration.

The findings of this systematic review provided specific support for core program features of workplace-based RTW programs. These core program features have been distilled to seven principles of return work (6): 1) the workplace has a strong commitment to health and safety which is demonstrated by the behaviours of the workplace parties, 2) the employer makes an offer of modified work (also known as work accommodation) to injured/ill workers so they can return early and safely to work activities suitable to their abilities, 3) RTW planners ensure that the plan supports the returning worker without disadvantaging co-workers and supervisors, 4) supervisors are trained in work disability prevention and included in RTW planning, 5) the employer makes an early and considerate contact with injured/ill workers, 6) someone has the responsibility to coordinate RTW, and 7) employers and health care providers communicate with each other about the workplace demands as needed, and with the worker's consent.

For peer review only - http://bmjopen.bmj.com/site/about/guidelines.xhtml

Franche recently reported on the factors associated with an offer of work accommodation in a cohort of more than 500 Ontario workers disabled by a musculoskeletal injury (7). Approximately 60% of workers received an offer of work accommodation in this cohort and workers were more likely to receive a work accommodation offer from their employer if the workplace was unionized or if the worker perceived strong disability prevention policies and practices in their workplace. In this large Ontario cohort, workplace factors were an important determinant of an offer of work accommodation. Workers who reported their workplace to be in the top third of a standardized measure of organizational policies and practices related to return-to-work (8-9) were three times more likely to receive an offer of work accommodation than workers who reported their workplace to be in the bottom third. Further work on this cohort has confirmed that organizational policies and practices in return-to-work are central factors in workplace disability prevention performance (10). Workers disabled by a musculoskeletal disorder who reported strong workplace organizational policies and practices in return-to-work were more likely to have returned to work at 6 months (OR=1.85; 95% CI 1.12-3.07) and at 12 months (OR=2.30; CI 1.31-4.04).

There are challenges in consistently administering a workplace disability management program (11-13). In many workplaces, an important proportion of disabled employees experience a variable and often undesirable return-to-work course including extended or intermittent work disability that results in significant individual, employer, and societal costs (13-15). Some of this inconsistency can be attributed to the sometimes competing goals of employers (e.g., minimize costs) and workers (e.g., recovery from illness), a lack of coordination and communication among work disability stakeholders (e.g., workers, health care providers, employers, and workers

For peer review only - http://bmjopen.bmj.com/site/about/guidelines.xhtml

compensation insurers), and gaps in the provision of adequate accommodations (14). In addition, supervisors and co-workers often have important responsibilities for managing different phases of the return-to-work process and their interaction with injured workers played an important role in determining work disability outcomes (16-18).

Of relevance to the objectives of this study, there is an important research literature on the factors that support successful implementation of disability management practices across organizations. An initiative in Denmark to systematically implement a national return-to-work program standard in 21 Danish municipalities identified substantial variation in disability outcomes following implementation (19-20). Evidence documented in a comprehensive process evaluation identified corresponding variation in the fidelity with which individual municipalities implemented five key dimensions of the return-to-work program and that weak implementation was associated with poor disability management outcomes (21). The current state of the research evidence concerning the effectiveness of organizational policies and practices to protect worker health points to the need for high-quality studies of the effectiveness of multi-component disability prevention programs.

The setting

The employer is one of Ontario's largest multi-site acute care community hospital systems with a $390 million annual operating budget and a staff complement of approximately 4300. Employees are represented by three unions.

For peer review only - http://bmjopen.bmj.com/site/about/guidelines.xhtml

The organization recognized limitations in the integrity of OHS policies and practices across the care sites in the system, including an under-developed disability management policy. There had been conflicts between management and representatives of workers over return-to-work practices, and the labour relations environment was frictional.

The Return-to-Work / Accommodation Policy

Over a 12 month period, the return-to-work / accommodation policy was developed jointly by the organization's management and union representatives, with contributions from not-for-profit external advisors. The evaluation research team did not participate in the development of return-to-work / accommodation policy. The policy was closely anchored to guidance provided by research evidence (5, 22-23), including an emphasis on early contact, the designation of disability case managers, the integration of supervisors in the development of return-to-work plans and the provision of education and training to managers and supervisors. A distinctive component of the policy was a specific role defined for union representatives as 'Return-to-Work Coordinators' (RTWC)(24). RTWCs participated in return-to-work planning meetings, along with the employee, the organization's disability case managers and the employee's supervisor. Details on the specific components of the return-to-work / accommodation policy have been published elsewhere (24). Implementation of the renewed return-to-work / accommodation policy commenced in January 2012.

The outcome objectives of the strengthened disability management policy were to reduce the incidence of work disability claims registered with the provincial workers' compensation authority by 25% and to reduce the total days of disability provided wage replacement benefits by 25% over the three year period 2012-2014.

For peer review only - http://bmjopen.bmj.com/site/about/guidelines.xhtml

Methods

Study Design

This study applied a mixed methods protocol consisting of two components. The process evaluation component had two elements: 1) a qualitative study of the perspectives of managers and supervisors concerning the fidelity of the implementation of the return to work / accommodation policy and, 2) the administration of two annual surveys of a sample of NHS staff who had returned to work following a disability episode. The outcome evaluation component applied a quasi-experimental design, comparing workers' compensation claim incidence and duration in the organization to a peer group of 29 large Ontario hospitals for a three year period prior to and following January 2012.

Process evaluation: supervisors and return-to-work coordinators

All managers and RTWCs who had been involved in a RTW episode in 2012 (N=104) were invited to participate in in-person semi-structured interviews. Interviews were completed with 21 managers and nine union RTWCs. Interviews followed a topic guide with general themes about the RTW policy: roles and responsibilities under the process; early contact practices; how the RTW plan was developed; how RTW is monitored; access to confidential information; and general overall assessment of the positive and negative elements of the program. A thematic analysis of interview transcripts was performed.

Process evaluation: Employees experiencing a disability episode

For peer review only - http://bmjopen.bmj.com/site/about/guidelines.xhtml

All employees who participated in a return-to-work plan following a disability episode in 2013 and 2014 were invited to complete an internet-administered 14 item questionnaire. In both years, the response rate was approximately 30%. The 14 item questionnaire asked respondents for their perception of the accommodation process using a five-point Likert scale (strongly agree to strongly disagree). Respondents were also invited to contribute free text responses on their overall experience, initial contact with the NHS's occupational health and safety staff, the RTW transition, and issues around health information confidentiality. Chi-square tests were applied to test for differences between 2013 and 2014 in the proportion of respondents who endorsed each of the 14 questionnaire items.

Outcome evaluation:

The outcome evaluation component applied a quasi-experimental design, comparing workers' compensation claim incidence and duration in the organization to a peer group of the 29 largest Ontario hospitals over the six year period 2009-2014.

Electronic abstracts of administrative records of lost-time and no lost-time compensation claims were obtained from the Workplace Safety & Insurance Board (WSIB), the publicly-administered, single-payer work disability insurer in Ontario. The outcome evaluation component of this study is based on two measures obtained from administrative data: 1) the incidence rate of lost-time and no lost-time claims and 2) the mean duration of lost-time disability episodes arising from work-related disorders. The incidence rates for lost-time and no lost-time claims were computed on an annual basis with denominator information of person years of employment obtained from WSIB insurance records of the organization and the peer

For peer review only - http://bmjopen.bmj.com/site/about/guidelines.xhtml

group of 29 large hospitals. The duration of disability was computed for each lost-time claim based on days of wage replacement benefits and the mean duration of disability, in days, computed for all claims on an annual basis. Confidence intervals (95%) for incidence rates and disability day rates were estimated under an assumption of a Poisson distribution. We estimated average annual percent change in incidence rates using negative binomial regression with the log of person-years of employment as an offset. We estimated annual rates of return-to-work at intervals of seven, fourteen, thirty and sixty days using piecewise exponential survival models in SAS PROC GENMOD and tested for differences in trends of return-to-work durations between the organization and the peer group (25). The outcome evaluation component of this study was designed to have sufficient power to detect a reduction in the incidence of workers' compensation claims from 75 per 1000 full-time equivalents (2009-2011) to 65 per 1000 full-time equivalents over the period 2012-2014.

Ethics

Ethical approval was obtained from the Health Sciences Research Ethics Board, University of Toronto (Protocol 28503). Written informed consent was obtained from all survey and interview participants.

For peer review only

Results

The implementation of the return-to-work / accommodation policy was the responsibility of the organization's occupational health and safety (OHS) department. The progress of the implementation was monitored by a labour-management steering committee, chaired by the organization's vice-president, human resources. Steering committee membership include union local leadership, OHS department staff and external members from a number of provincial labour unions. The steering committee met 4-6 times per year during the three year implementation period documented in this study. At the invitation of the labour and employer members of the organization, the research team was also a member of the steering committee and periodically provided reports on aspects of the process and outcomes of the policy implementation.

Process evaluation component

Both qualitative and quantitative measures of the implementation process were predominantly positive. Supervisors, managers and RTWCs who participated in semi-structured interviews in 2013 noted a number of achievements (24). Informants offered the view that occupational health and safety practice had improved since the implementation of the policy and spoke of the clear internal communication around the return-to-work / accommodation policy to all levels of staff in the organization. Informants also perceived a renewed emphasis on returning disabled workers to their pre-injury positions as quickly as possible and that the staff in the occupational health and safety department were well respected. There was recognition that the quality and consistency of disability management practices were improving, that the collaboration between the employer and the labour unions was strengthened and that the return-to-work planning

For peer review only - http://bmjopen.bmj.com/site/about/guidelines.xhtml

meeting was an important opportunity for establishing clarity for the goals of individual return-to-work plans.

Managers, supervisors and return-to-work coordinators also identified a number of opportunities for improvement. Informants encouraged a greater emphasis on timely access to information about the status of individual disability episodes, and identified a number of opportunities to improve the consistency of case management across disability episodes. Informants also described a number of areas where the roles and responsibilities return-to-work coordinators and supervisors were ambiguous and emphasized the importance of providing orientation to newly-appointed personnel. Interview participants also acknowledged that the OHS department had a high case load and constrained resources.

For peer review only

Employees experiencing a disability episode

Approximately 30% of employees who participated in a return to work plan accepted the invitation to complete an internet-administered 14 item questionnaire. The majority of respondents who returned to work in 2013 (N=78) and in 2014 (N=54) reported positive assessments of the return-to-work process (Table 1). Employees gave strong endorsement to the purpose and outcome of the return-to-work planning meeting and expressed satisfaction with the protection of personal health information. Respondents in 2014 reported stronger endorsement of a number of program dimensions compared to respondents in 2013. For example, a higher proportion of respondents in 2014 were satisfied with modified duty arrangements and a smaller proportion of respondents in 2014 reported delays in arranging the initial return-to-work planning meeting. There were no significant differences in perceptions of the return-to-work

For peer review only - http://bmjopen.bmj.com/site/about/guidelines.xhtml

process between respondents whose disability was attributed to a work-related cause, and respondents whose disability was attributed to a non-occupational cause.

Free text responses provided by survey respondents were approximately balanced between positive and negative statements. Themes that were emphasized in the free text responses included the importance of supportive, compassionate and respectful interactions with OHS staff, the importance of support from managers and supervisors and the value of efficient and timely communication with OHS staff. Respondents also emphasized the value of monitoring the progress of accommodations over the course of the disability episode and the importance of aligning modified duties to clinical recommendations.

<u>Outcome evaluation component</u>

Over the six year observation period there were 1,916 total compensation claims in the organization, and 29,974 compensation claims in the comparison group of 29 large hospitals (Table 2). The incidence rates for lost-time claims and for no lost-time claims were substantially higher in the organization relative to the comparison group. In both the organization and the comparison group, the decline in the incidence of lost-time claims between the 2009-2011 period and the 2012-2014 period was statistically significant. The annual percent change in the lost-time claim incidence rate in the organization was -5.6 (95% CI: -9.9, -1.1) comparable to the annual percent change in the comparison group: -6.2 (-7.2, -5.3).

Disability durations also declined in the organization, from a mean of 19.4 days (16.5, 22.3) in the pre-intervention period to 10.9 days (8.7, 13.2) in the post-intervention period (Table 3).

For peer review only - http://bmjopen.bmj.com/site/about/guidelines.xhtml

Reductions in disability durations were also observed in the comparison group: from a mean of 13.5 days (12.9, 14.1) in the 2009-2011 period to 10.5 days (9.9, 11.1) in the 2012-2014 period.

In the pre-intervention period, the proportion of disability episodes in the organization that had returned to work at seven days, 30 days and 60 days was substantially lower than observed outcomes in the peer group. As depicted in Figure 1, the proportion of disability episodes that had returned to work at seven days improved in both the organization and the comparison group over the six year observation period, with a stronger improvement in the organization. A test for difference in the trend of improvement between the organization and the comparison group approached statistical significance ($p=.07$).

For peer review only

Discussion

This study has described the process and outcomes of a three year effort to implement a strengthened disability management policy in a large healthcare employer. Both the process and outcome findings of this study document a successful implementation of the strengthened policy. Managers, supervisors and return-to-work coordinators broadly endorsed the strengthened policy. Employees who returned to work following a disability episode provided predominantly positive assessments across all dimensions of the disability management process. The annual percent reduction in lost-time claims in the organization was similar to the reductions observed in a peer group of 29 hospitals over a six year period, consistent with a broad trend of a decline in the incidence of work-related injury and illness in all economic sectors in this jurisdiction (26). Independent of this trend in declining incidence, the implementation of the organization's strengthened return-to-work policy was associated with greater improvements in disability durations than observed in the peer group.

Despite the broad consensus that workplace polices and practices are a central determinant of return-to-work outcomes, the literature on the implementation of disability management programs in individual workplaces is limited and represents a research gap (2-3). This study contributes to addressing this research gap. While the observational study was not suitable for determining which elements of the strengthened disability management policy were most responsible for the improvements in disability durations, the favourable process and outcome measures reported in this study indicate that it is feasible to successfully implement a moderately complex multi-component disability management policy. From the perspective of employees who returned to work following a disability episode, it appears that all elements of the return-to-

For peer review only - http://bmjopen.bmj.com/site/about/guidelines.xhtml

work process were meaningful, including early contact, the process and outcome of the return-to-work planning meeting and the support of supervisors and union representatives in the transition back to work. The collaborative model adopted by this organization, where representatives of a worker's union participate in return-to-work planning, appears to have functioned effectively. RTW coordinators and disability management staff developed respect for the consistency and transparency of the collaborative process. And we note there was continuity and consistency in the organizational leadership supporting this initiative.

The evaluation design combining qualitative and quantitative methods to document the process and outcome of the policy implementation was a strength of this study. The inclusion of a comparison group of peer hospitals operating in the same geographic jurisdiction provided an accurate calibration of the magnitude of change achieved by the organization.

We would briefly note the following limitations. Although the evaluation design did include a quasi-experimental component, the study has reported on the process and outcome of change in a single organization. Stronger insights into the facilitators and barriers of organizational improvement in disability management practices would be available from evaluations of implementation process and outcome across multiple workplaces, where the attributes of organizations that successfully accomplish the program objective can be compared to organizations that are less successful (21, 27-28). While this study has compared the incidence and durations of disability episodes arising from work-related conditions between the organization and a large hospital peer-group, we were unable to acquire similar administrative

For peer review only - http://bmjopen.bmj.com/site/about/guidelines.xhtml

records of work disability incidence and duration for disability episodes not attributed to work exposures.

The organizational change initiative reported in this study was designed jointly by labour and management representatives within a large Ontario public sector employer, with technical assistance provided by not-for-profit external agencies with expertise in workplace quality improvement initiatives. This organization change initiative was accomplished with the technical and human resources available to the employer and did not require significant external resources. Relative to the outcome objectives established at the initiation of this quality improvement initiative, the organization accomplished a reduction of approximately 15% in the incidence of work disability claims and achieved an approximate 50% reduction in average disability episode durations over the three year period following the implementation of the strengthen disability management policy. Replication of the collaborative model describes in this study may be considered in other healthcare settings in this jurisdiction.

For peer review only

For peer review only - http://bmjopen.bmj.com/site/about/guidelines.xhtml

Contributorship statement

C Mustard conceptualized and designed the study, interpreted the data and wrote the first draft of the paper. K Skivington, M Lifshen, A Chambers and M Lay contributed to data collection and data interpretation. J Etches contributed to the analysis of the study data and provided interpretation. All authors contributed to the review and revision of the paper.

Competing interests

The authors declare no competing interests.

Funding

Funding support for the study reported in this paper was provided by the Canadian Institutes of Health Research (Grant No. 123365). KS is funded by the Medical Research Council (MC_PC_13027 & MC_UU_12017/15). The Institute for Work & Health operates with support from the Government of Ontario.

Data sharing statement

No additional data available.

For peer review only

References

1. Frank J, Cullen K. Preventing injury, illness and disability at work. Scandinavian Journal of Work, Environment and Health. 2006;32(2):160-7.

2. Viikari-Juntura E, Burdorf A. Return to work and job retention – increasingly important outcomes in occupational health research. Scand J Work Environ Health. 2011;37(2):81-84.

3. Vanderbroeck S, Verjans M, Lambreghts C, Godderis L. Research review on rehabilitation and return to work. Luxembourg: European Agency for Safety and Health at Work; 2016. doi: 978-92-9240-924-1

4. Main CJ, Shaw WS; Hopkinton Conference Working Group on Workplace Disability Prevention. Employer Policies and Practices to Manage and Prevent Disability: Conclusion to the Special Issue. J Occup Rehabil. 2016 Dec;26(4):490-498.

5. Franche RL, Cullen K, Clarke J, Irvin E, Sinclair S, Frank J. Workplace-based return-to-work interventions: a systematic review of the quantitative literature. Journal of Occupational Rehabilitation. 2005;15(4):607-31.

6. Institute for Work & Health. Seven 'principles' for successful return to work. Available from: http://www.iwh.on.ca/files/seven_principles_rtw_2007.pdf.

7. Franche RL, Severin C, Hogg-Johnson S, Lee H, Côté P, Krause N. Multivariate analysis of factors associated with early offer and acceptance of a work accommodation following an occupational musculoskeletal injury. Journal of Occupational and Environmental Medicine. 2009 Aug;51:969-983.

8. Williams RM, Westmorland MG, Shannon HS, Amick BC. Disability management practices in Ontario health care workplaces. J Occup Rehabil. 2007 Mar;17(1):153-65.

9. Williams RM, Westmorland MG, Shannon HS, Rasheed F, Amick BC. Disability management practices in education, hotel/motel and health care workplaces. Am J Ind Med 2005;47:217–26.

10. Amick BC, Lee H, Hogg-Johnson S, Katz JN, Brouwer S, Franche RL, Bultmann U. How do organizational policies and practices affect return-to-work and work role functioning following a musculoskeletal injury. J Occup Rehabil. Epub 2016 Sept 21. 2016. doi: 10.1007/s10926-016-9668-8

11. Costa-Black KM, Feuerstein M, Loisel P. Work Disability Models: Past and Present. In: Handbook of Work Disability. New York: Springer; 2013. p. 71-93.

12. Amick BC, Habeck R, Hunt A, Fossel A, Chapin A, Keller R, et al. Measuring the impact of organizational behaviors on work disability prevention and management. Journal of Occupational Rehabilitation. 2000;10(1):21-38.

13. MacEachen E, Kosny A, Ferrier S, Chambers L. The "toxic dose" of system problems: why some injured workers don't return to work as expected. Journal of Occupational Rehabilitation. 2010;20(3):349-66.

For peer review only - http://bmjopen.bmj.com/site/about/guidelines.xhtml

14. MacEachen E, Kosny A, Ferrier S. Unexpected barriers in return to work: lessons learned from injured worker peer support groups. Work: A Journal of Prevention, Assessment and Rehabilitation. 2007;29(2):155-64.

15. Jetha A, Pransky G, Fish J, Hettinger LJ. Return-to-work within a complex and dynamic organizational work disability system. J Occup Rehabil. 2016 Sep;26(3):276-85. doi: 10.1007/s10926-015-9613-2.

16. Besen E, Young AE, Shaw WS. Returning to work following low back pain: towards a model of individual psychosocial factors. Journal Occup Rehabil. 2014:1-13.

17. Tjulin Å, MacEachen E, Ekberg K. Exploring workplace actors experiences of the social organization of return-to-work. Journal Occup Rehabil. 2010;20(3):311-21.

18. Linton S, Boersma K, Traczyk M, Shaw W, Nicholas M. Early workplace communication and problem solving to prevent work disability: results of a randomized controlled trail among high-risk workers and their supervisors. J Occup Rehabil. 2016;26:150-159.

19. Aust B, Helverskov T, Nielsen MB, Bjorner JB, Rugulies R, Nielsen K, et al. The Danish national return-to-work program – aims, content, and design of the process and effect evaluation. Scand J Work Environ Health. 2012 Mar;38(2):120-33. doi: 10.5271/sjweh.3272.

20. Nielsen MB, Vinsløv Hansen J, Aust B, Tverborgvik T, Thomsen BL, et al. A multisite randomized controlled trial on time to self-support among sickness absence beneficiaries. The Danish national return-to-work programme. Eur J Public Health. 2015 Feb;25(1):96-102. doi: 10.1093/eurpub/cku016.

21. Aust B, Nielsen MB, Grundtvig G, Buchardt HL, Ferm L, Andersen I, et al. Implementation of the Danish return-to-work program: process evaluation of a trial in 21 Danish municipalities. Scand J Work Environ Health. 2015 Nov;41(6):529-41. doi:10.5271/sjweh.3528.

22. Pranksy G, Shaw W, Loisel P, Hong N.H, Désorcy B. Development and validation of competencies for Return to Work Coordinators. J Occup Rehabil. 2010;20:41-48.

23. Gensby U, Labriola M, Irvin E, Amick BC, Lund T. A classification of components of workplace disability management programs: results from a systematic review. J Occup Rehabil. 2014 Jun;24(2):220-41. doi: 10.1007/s10926-013-9437-x.

24. Skivington K, Lifshen M, Mustard CA. Implementing a collaborative return-to-work programme: lessons from a case study of a large employer in Canada. Work. 2016 Nov 22;55(3):613-624. doi: 10.3233/WOR-162437

25. Allison D. Survival analysis using SAS: A practical guide. 2nd ed. SAS Institute; 2010.

26. Chambers A, Ibrahim S, Etches J, Mustard C. Diverging trends in the incidence of occupational and nonoccupational injury in Ontario, 2004-2011. Am J Public Health. 2015 Feb;105(2):338-43. doi: 10.2105/AJPH.2014.302223.

27. Fassier JB, Durand MJ, Caillard JF, Roquelaure Y, Loisel P. Results of a feasibility study: barriers and facilitators in implementing the Sherbrooke model in France. Scand J Work Environ Health. 2015 May 1;41(3):223-33. doi:10.5271/sjweh.3489.

For peer review only - http://bmjopen.bmj.com/site/about/guidelines.xhtml

28. Chambers A, Mustard CA, Holness LD, Nichol K, Breslin CF. Barriers to the adoption of safety-engineered needles following a regulatory standard: Lessons learned from three acute care hospitals. Healthcare Policy. 2015;11(1):90-101.

For peer review only

For peer review only - http://bmjopen.bmj.com/site/about/guidelines.xhtml

Table 1

Returned-to-work employees' perceptions of the disability management process

Five point likert scale (strongly disagree to strongly agree)

Highlighted items indicate significant difference (improvement) between 2013 and 2014 surveys

	Percent of employees agreeing with statement		
	**2013** (n=78)	**2014** (n=54)	Chi sq
**Supported through the process:**			
My supervisor/manager supported my transition back to work	70%	76%	0.49
My coworkers supported by transition back to work	76%	78%	0.78
My absence and my return-to-work experience was in keeping with my employer's core values of compassion, professionalism and respect	53%	67%	0.11
**Satisfaction with the process:**			
Overall, I was satisfied with my union's support in my return-to-work	72%	69%	0.69
Overall, I was satisfied with the employer's support in my return-to-work	58%	68%	0.21
I was satisfied with arrangement to modify my job duties and/or work hours during the transition back to full regular duties	60%	81%	0.01
**Confidence about the process:**			
Timeliness			
There were delays in arranging my return-to-work planning meeting	42%	28%	0.09
Documentation from my healthcare provider supported the timeliness of my return-to-work	83%	91%	0.22
I was contacted shortly after my absence began by the Occupational Health and Safety Department	76%	80%	0.60
The return-to-work meeting			
I understood the purpose of the return-to-work planning meeting	87%	87%	0.98
The return-to-work planning meeting identified options to modify or redesign my regular work and/or hours	67%	83%	0.03
I was an active participant in the return-to-work planning meeting	79%	82%	0.77
Planning my return-to-work was a team effort	64%	80%	0.05
Confidentiality			
Confidential information about my health was protected	72%	85%	0.07

For peer review only

For peer review only - http://bmjopen.bmj.com/site/about/guidelines.xhtml

Figure 1

Trend in disability benefit duration, seven days or less

Organization compared to 29 peer hospitals, Ontario 2009-2014

Work-related lost-time compensation claims

For peer review only

For peer review only - http://bmjopen.bmj.com/site/about/guidelines.xhtml

Table 2
Incidence of compensation claims, Organization compared to 29 peer hospitals, 2009-2014

	2009-2011				2012-2014			
	N	FTE	Rate/1000	95% CI (1)	N	FTE	Rate/1000	95% CI (1)
**Lost-time claims**								
Organization	362	12,062	30.0	27.1 - 33.3	287	12,849	22.3	19.9 - 25.1
Peer Hospitals	4,983	357,104	14.0	13.6 - 14.3	3,986	379,150	10.5	10.2 - 10.8
**No Lost-time claims**								
Organization	631	12,062	52.3	48.4 - 56.6	636	12,849	49.5	45.8 - 53.5
Peer Hospitals	9,835	357,104	27.5	27.0 - 28.1	11,170	379,150	29.5	28.9 - 30.0
**Total claims**								
Organization	993	12,062	82.3	77.4 - 87.6	923	12,849	71.8	67.3 - 76.6
Peer Hospitals	14,818	357,104	41.5	40.8 - 42.2	15,156	379,150	40.0	39.3 - 40.6

(1) The CI assumes a Poisson distribution in the numerator.

For peer review only - http://bmjopen.bmj.com/site/about/guidelines.xhtml

Table 3

Lost-time compensation claim benefit duration, Organization compared to 29 peer hospitals, 2009-2014

Average benefit days per claim, first 90 days post-injury

	2009-2011			2012-2014		
	Number of Claims	Average benefit days	95% CI	Number of Claims	Average benefit days	95% CI
**Lost-time claims**						
Organization	362	19.4	16.5 - 22.3	287	10.9	8.7 - 13.2
Peer Hospitals	4,983	13.5	12.9 - 14.1	3,986	10.5	9.9 - 11.1

peer review only

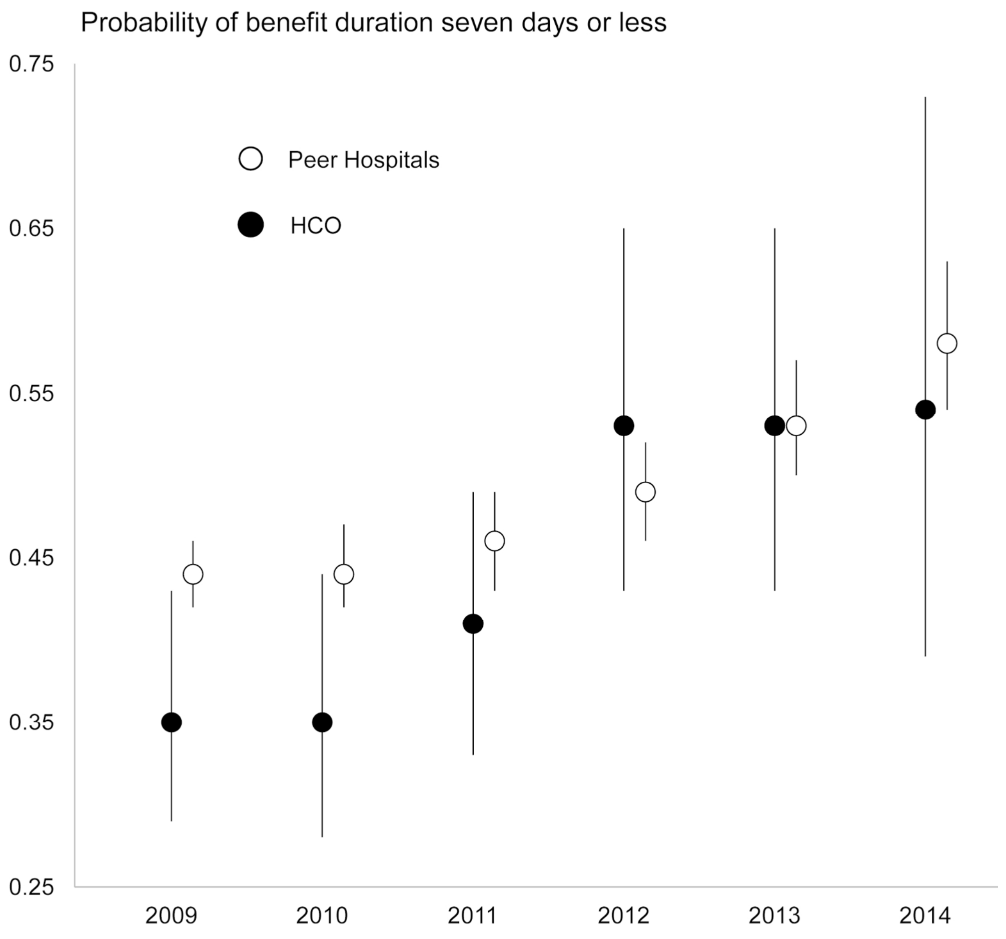

Figure 1
Trend in disability benefit duration, seven days or less
Organization compared to 29 peer hospitals, Ontario 2009-2014
Work-related lost-time compensation claims

173x160mm (300 x 300 DPI)
